# The effect of Chinese herbs and its effective components on coronary heart disease through PPARs-PGC1α pathway

**DOI:** 10.1186/s12906-016-1496-z

**Published:** 2016-12-12

**Authors:** Qiyan Wang, Chun Li, Qian Zhang, Yuanyuan Wang, Tianjiao Shi, Linghui Lu, Yi Zhang, Yong Wang, Wei Wang

**Affiliations:** 1 School of Life Sciences, Beijing University of Chinese Medicine, Beijing, 100029 China; 2 Modern Research Center for Traditional Chinese Medicine,School of Chinese Materia Medica, Beijing University of Chinese Medicine, Beijing, 100102 China; 3 School of Basic Medical Science, Beijing University of Chinese Medicine, Beijing, 100029 China

**Keywords:** Components, Coronary heart disease, PPARs-PGC1α pathway, DanQi pill

## Abstract

**Background:**

DanQi pill (DQP) is prescribed widely in China and has definite cardioprotective effect on coronary heart disease. Our previous studies proved that DQP could effectively regulate plasma levels of high density lipoprotein (HDL) and low density lipoprotein (LDL). However, the regulatory mechanisms of DQP and its major components Salvianolic acids and Panax notoginseng saponins (DS) on lipid metabolism disorders haven’t been comprehensively studied so far.

**Methods:**

Rat model of coronary heart disease was induced by left anterior descending (LAD) artery ligation operations. Rats were divided into sham, model, DQP treated, DS treated and positive drug (clofibrate) treated groups. At 28 days after surgery, cardiac functions were assessed by echocardiography. Expressions of transcription factors and key molecules in energy metabolism pathway were measured by reverse transcriptase polymerase chain reaction or western blotting.

**Results:**

In ischemic heart model, cardiac functions were severely injured but improved by treatments of DQP and DS. Expression of LPL was down-regulated in model group. Both DQP and DS could up-regulate the mRNA expression of LPL. Membrane proteins involved in lipid transport and uptake, such as FABP4 and CPT-1A, were down-regulated in ischemic heart tissues. Treatment with DQP and DS regulated lipid metabolisms by up-regulating expressions of FABP4 and CPT-1A. DQP and DS also suppressed expression of cytochrome P450. Furthermore, transcriptional factors, such as PPARα, PPARγ, RXRA and PGC-1α, were down-regulated in ischemic model group. DQP and DS could up-regulate expressions of these factors. However, DS showed a better efficacy than DQP on PGC-1α, a coactivator of PPARs. Key molecules in signaling pathways such as AKT1/2, ERK and PI3K were also regulated by DQP and DS simultaneously.

**Conclusions:**

Salvianolic acids and Panax notoginseng are the major effective components of DanQi pill in improving lipid metabolism in ischemic heart model. The effects may be mediated by regulating transcriptional factors such as PPARs, RXRA and PGC-1α.

## Background

Coronary heart disease (CHD) induced by atherosclerosis remains one of the major threats to people’s health and one of the main causes of death, despite our better understanding of the pathophysiology of CHD and advances in treatment methods [1]. In developing countries, the incidence of CHD has been rising in recent years [2]. It’s of paramount importance to develop novel therapies for CHD so as to reduce the economic and health burden caused by it.

Managements of CHD include life style changes, medication and surgical procedures [3, 4]. Antihypertensive drugs such as ACEIs and lipid lowering drugs are the most commonly prescribed medications. Traditional Chinese medicine (TCM) has also been used to treat CHD for a long period of time. DanQi Pill (DQP), composed of Radix Salvia Miltiorrhiza (Danshen) and Panax notoginseng (Sanqi), is among the most commonly prescribed TCM [5] Salvianolic acids are the water soluble compounds extracted from Radix Salvia Miltiorrhizae [6, 7], and Panax notoginseng saponins are the major active components of Panax notogeinseng [8, 9]. Exploration of the pharmacological mechanisms of DQP will provide insight into the treatment of CHD and development of new drugs.

Our previous studies proved that DQP could effectively regulate plasma levels of high density lipoprotein (HDL) and low density lipoprotein (LDL) in rat models of CHD [9]. DQP could also modulate cardiac energy metabolism that has been disturbed under ischemic conditions [10]. In ischemic heart disease, overall mitochondrial oxidative catabolism decreases while reliance on anaerobic glycolysis pathways is increased [11, 12]. Fatty acids oxidation was effectively improved by DQP, as was shown by up-regulated fatty acids transportation, uptake and metabolism. DQP exerts cardioprotective effect by improving energy supply of myocytes. However, the upstream transcriptional regulatory effects of DQP haven’t been studied yet. Furthermore, the effects of compound formula (DQP) and major components (Salvianolic acids and Panax notoginseng saponins, DS) haven’t been evaluated yet.

The peroxisome proliferator-activated receptor family (PPARα, β/δ and γ) of nuclear receptor transcription factors is an important regulator of cardiac metabolism [12]. The PPARs control myocardial metabolism by transcriptionally regulating genes encoding enzymes involved in fatty acid and glucose utilization [13, 14]. Peroxisome proliferator-activated receptor gamma coactivator-1α (PGC-1α) is transcriptional coactivator of the PPARs and is a key player in the control of myocardial metabolism. Activation of PGC-1α drives a strong induction of PPARs target genes encoding fatty acid oxidation enzymes [15]. In this study, we investigated the effects of DQP and the extracts of DQP (DS, Panax notoginseng saponins and Salvianolic acids) on PPARs and PGC-1α. The target genes of these transcriptional regulators involved in energy metabolism and down-stream signaling pathways were also detected. Rat model of ischemic coronary heart disease was applied in this work. This study will provide further insight into the mechanisms of traditional Chinese medicine in the management of CHD.

## Methods

### Animal groupings and induction of myocardial infarction

One hundred Sprague-Dawley (SD) male rats, with weight of 220 ± 10 g, were randomly divided into five groups: sham-operated, model, positive control drug (clofibrate) treated, solutions of DS treated and DQP treated groups. All the rats were purchased from Beijing Vital River Laboratory Animal Technology Co. Ltd, with the license number of SCXK2010 ~ 2011. The animal experiments were approved by the Animal Care Committee of Beijing University of Chinese Medicine. The rats were fed with regular diet for one week before surgery. Rats in the control group received sham operation while rats in the other groups underwent left anterior descending (LAD) artery ligation surgeries which induced models of ischemic heart disease. The operational procedures have been described in our previous studies [10, 16]. Briefly, left thoracotomies were performed after rats were anaesthetized by 1% pentobarbital sodium at the dosage of 50 mg/kg *via* intraperitoneal injection. LAD coronary arteries were then ligated with a 5–0 polypropylene suture. The thorax was closed after ligation. Rats in the sham group received the same procedures except that the coronary arteries were not ligated. From the second day after surgery, Rats were randomly divided into five groups: sham group fed with normal diet, model group, clofibrate group (positive control), DQP group and DS group fed with high fat diet for 28 days. Rats in the DQP group were treated with DQP aqueous solution (Tongren tang, Beijing, China, Series: 6128006) at the dosage of 1.5 mg/kg per day via oral gavage for 28 consecutive days [9]. In DS group, rats received solutions of salvianolic acid (SA) and Panax Notoginseng Saponins (PNS) at the dosage of 7.5 μg/kg and 82.5 μg/kg respectively. The dosage of SA and PNS were given according to their percentage contained in DQP. Rats in the positive control group received clofibrate aqueous solution at the dosage of 0.3 g/kg as reported previously. Animals in the sham and model groups were given a gavage of normal saline water for 28 days. The mortality rate of surgery was 22%. After surgery, 19(95%), 15(75%), 14(70%), 16(80%) and 14(70%) rats survived in sham, model, positive control drug treated, DS treated and DQP treated groups, respectively.

### Measurement of cardiac functions

Echocardiography was applied to assess the cardiac functions at 28d after surgery using a Vevo 2100 (VisualSonics Inc, Toronto, Ontario, Canada). A PST 65A sector scanner (8-MHz probe) was employed, which generates two-dimensional images at a frame rate of 300 to 500 frames/s. The parameters of heart functions include: left ventricular end-systolic diameter (LVESd), left ventricular end-diastolic diameter (LVEDd), left ventricular end-diastolic volume (LVEDv), left ventricular end-systolic volume (LEVSv), ejection fraction (EF) and fractional shortening (FS). EF was calculated as follows: EF % = [(LVEDv ‐ LVESv)/LVEDv] × 100%. FS was calculated using the equation: [(LVEDd ‐ LVESd)/LVEDd] × 100%.

### Evaluation of mRNA expressions of key molecules involved in lipid metabolism pathway

The mRNA expressions of Lipoprotein Lipase (LPL), cluster of differentiation 36 (CD36) and ATP-binding cassette transporter (ABCA1) were determined by reverse transcriptase polymerase chain reaction (RT-PCR). Total RNA was extracted using TRIzol Reagent (Gibco-BRL, Paisley, UK). The concentration of RNA was measured using Nano Drop 2000 (Thermo Scientific, USA), and the RNA was then transcribed to cDNA using the RevertAidTM First Stand cDNA Synthesis kit (Fermentas, LT). The reaction volume was 20 μl, containing 2 μl cDNA, 0.5 μl forward and 0.5 μl reverse primers, 10 μl Rox and 7 μl DEPC. For cDNAs analysis of LPL, ABCA1, CD36 and GAPDH, PCR conditions were set as follows: 15 s at 95 °C for denaturation, and 1 min at 55 °C for annealing and extension. 40 cycles were run for each gene. The sequences of the primers were shown at Table 1. Quantities of each gene were normalized to quantity of GAPDH mRNA.

**Table 1:** Nucleotide sequences of primers used in real-time PCR

Gene(accession no.)	Primers	Nucleotiede sequences5′-3′	Size(bp)	Temp(°C)
LPL	Forward	CGCTCCATCCATCTCTTC	18	57.3
	Reverse	GGCTCTGACCTTGTTGAT	18	55
ABCA1	Forward	GTGGTGTTCTTCCTCGTTA	19	55.4
	Reverse	CTTCCGCTTCCTTCTGTAG	19	57.6
CD36	Forward	GGCCCTTACACATACAGAGT	20	55.8
	Reverse	CCACAGCCAGATTGAGAA	18	55

### Measurement of key proteins in energy metabolism pathways by western blot (WB)

Left ventricle homogenates were prepared for the analysis of protein levels. Briefly, the cardiac tissue was homogenized in RIPA buffer (50 mM Tris-HCl Ph7.4, 150 mM NaCl, 1% NP-40, 0.1%SDS) and total protein was extracted from this homogenate. The protein concentration in each sample extract was measured by a protein assay kit (Beijing PuLilai Gene Technology Co., Ltd, Beijing, China, lot number: P1511). Equal amounts of protein extracts (20 μg) were separated by 12.5% sodium dodecyl sulphate(SDS)-polyacrylamide gel electrophoresis (Bio-Rad, CA, U.S.A.) and transferred to nitrocellulose membranes electrophoretically (semidry transfer). Membranes were blocked with 5% non-fat dry milk in Tris-buffered saline (20 mM Tris, pH 7.6, 137 mM NaCl) with 0.1% Tween 20, washed, and then incubated with primary antibody. Primary antibodies employed included: goat polyclonal anti-glyceraldehyde-3-phosphate dehydrogenase(GAPDH) and anti- Peroxisome proliferator-activated receptor α (PPARα,ab8934), Peroxisome proliferator-activated receptor γ (PPARγ, SC7273), fatty acid binding protein (FABP, ab174673), Apolipoprotein A-I(ApoA-I,ab334707), Carnitine palmitoyltransferase 1A(CPT-1A, ab128568), 3-hydroxy-3methyl-glutaryl-CoA reductase (HMGCR(H-300), SC-33827,SANTA), P450(Anti-Cytochrome P450 Reductase antibody, ab13513), peroxisome proliferator activated receptor coactivator 1 alpha(PGC-1α, ab54481), AKT1/2(ab6067), Phosphatidylinositol 3-kinase(PI3K, ab151549) and extracellular signal-regulated kinase(ERK1/2, ab54230). The primary antibody was firstly incubated, and then the secondary antibodies (Santa Cruz Biotechnology Inc., CA, U.S.A.) was added. After being exposed to chemiluminescence developing agents, the protein levels and GAPDH in each sample were evaluated. The gel was scanned and the band densities were quantified. The band densities of proteins were normalized by the GAPDH band densities to determine their concentrations.

### Statistical analysis

Data were presented as mean ± standard deviation. Differences among groups were analyzed by one-way analysis of variance (ANOVA) test. P values of smaller than 0.05 were considered as statistically significant. All statistical analyses were carried out by SPSS 17.0 software.

## Results

### Effects of DQP and DS on cardiac functions

Twenty eight days after surgery, ultrasonography was performed to evaluate heart functions in different groups of animals. LVEDd and LVEDs increased significantly in the model group compared with those in the sham group, indicating that hypertrophy had developed as a result of ischemia in rats that underwent ligation surgery. EF and FS were reduced by 39.91% (*P* < 0.05) and 44.6% (*P* < 0.05) in the model group compared with those in the sham group, suggesting that cardiac functions were severely impaired in the model rats. After treatments with DQP, LVEDd and LVEDs were down-regulated by 32.14% (*P* < 0.05) and 28% (*P* > 0.05), and EF and FS were up-regulated by 15.03% (*P* < 0.05) and 63.40% (*P* < 0.05) in the DQP treatment group compared with those in the model group. In DS treatment group, EF and FS value were also improved compared with those in the model group (Table 2). These data indicated that DQP and DS could exert anti-hypertrophy and cardioprotective effects in ischemic heart.

**Table 2:** Indicators of heart functions tested by Echocardiography in different groups of rats

	Sham	Model	Positive drug	DQP	DS
LVEDd(cm)	0.64 ± 0.05^*^	1.12 ± 0.21	1.08 ± 1.68	0.76 ± 0.064^*^	0.83 ± 0.18
LVEDs(cm)	0.41 ± 0.02^**^	0.75 ± 0.05	0.48 ± 0.08^*^	0.54 ± 0.080	0.64 ± 0.11
EF	0.68 ± 0.06^**^	0.41 ± 0.03	0.46 ± 0.06	0.47 ± 0.03^*^	0.53 ± 0.01^*^
FS(%)	37.39 ± 5.19^*^	20.71 ± 1.68	21.70 ± 5.78	33.84 ± 6.90^*^	36.43 ± 8.76^*^

^*^v.s. model group, *P* < 0.05, ^**^v.s. model group, *P* < 0.01

### Effects of DQP and DS on proteins associated with lipid metabolism

**Fig. 1:**
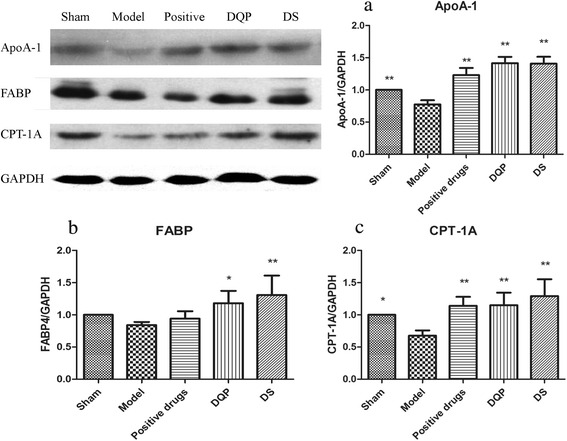
Expressions of ApoA-1, FABP4 and CPT-1A in five groups of rats. Western blot showed that compared with model group, expressions of these three molecules were significantly up-regulated towards normal levels in DQP and DS groups. **a** Semi-quantitative expressions of ApoA-1 in different groups. **b** Semi-quantitative expressions of FABP4 in different groups. **c** Semi-quantitative expressions ofCTP-1A in different groups. *n* = 6, * v.s. model group, *P* < 0.05; ** v.s. model group, *P* < 0.01

Expressions of lipoproteins and proteins involved in lipid metabolism were detected. ApoA-I, the major protein component of HDL, can promote cholesterol reversal transport by activating lecithin cholesterolacyltransferase (LCAT) [17]. Level of ApoA-I in model group (0.77 ± 0.07) was reduced compared as that in the sham group (1.00 ± 0.00) (*P* < 0.01). DQP and DS could increase plasma levels of ApoA-1 (1.41 ± 0.11, 1.40 ± 0.11) (*P* < 0.01), promoting transportation of lipids from tissues to the liver for excretion (Fig. 1). Lipoprotein lipase (LPL) hydrolyzes triglycerides in lipoproteins and promotes cellular uptake of chylomicron remnants and cholesterol-rich lipoproteins [18]. Transcription level of LPL in model group was reduced. After treatment with DQP and DS, LPL was up-regulated significantly (Table 3). ATP-binding cassette transporter (ABCA1), which is involved in cholesterol efflux pumping and formation of HDL, was down-regulated in model group compared with that in the sham group. Expression of ABCA1 was up-regulated after treatment with DS (Table 3).

**Table 3:** mRNA expressions of LPL, CD36 and ABCA1 in each groups

	Sham	Model	Positive	DQP	DS
LPL	3.56 ± 2.75^*^	0.15 ± 0.07	1.75 ± 0.76^**^	1.05 ± 0.60^*^	1.45 ± 0.81^*^
ABCA1	0.84 ± 0.60^*^	0.27 ± 0.21	1.36 ± 0.56^*^	1.30 ± 0.95	1.51 ± 0.67^**^
CD36	1.27 ± 0.97^*^	0.76 ± 0.46	0.84 ± 1.03	0.77 ± 0.30	0.94 ± 0.57

*v.s. model group, *P < 0.05; **P < 0.01

Membrane molecules that are involved in lipid uptake into myocardial cells and mitochondria for oxidation were also detected. Key membrane proteins includes: CD36, fatty acids binding protein 4(FABP4), and carnitine palmitoyl transferase 1A (CPT-1A). Expression of CD36 and CPT-1A were significantly reduced in model group compared with those in the sham group (*P* < 0.05). Expression of FABP4 was also reduced in ischemic heart tissue, but the reduction showed no statistical significance. After treatment with DQP and DS for 28 days, expressions of both FABP4 and CPT-1A were up-regulated significantly, indicating that expressions of these molecules were activated and uptake of lipids into cardiac cell were improved (Table 3 and Fig. 1). No statistical differences were observed between effects of the DQP and DS.

### Effects of DQP and DS on cholesterol synthesis and metabolism

**Fig. 2:**
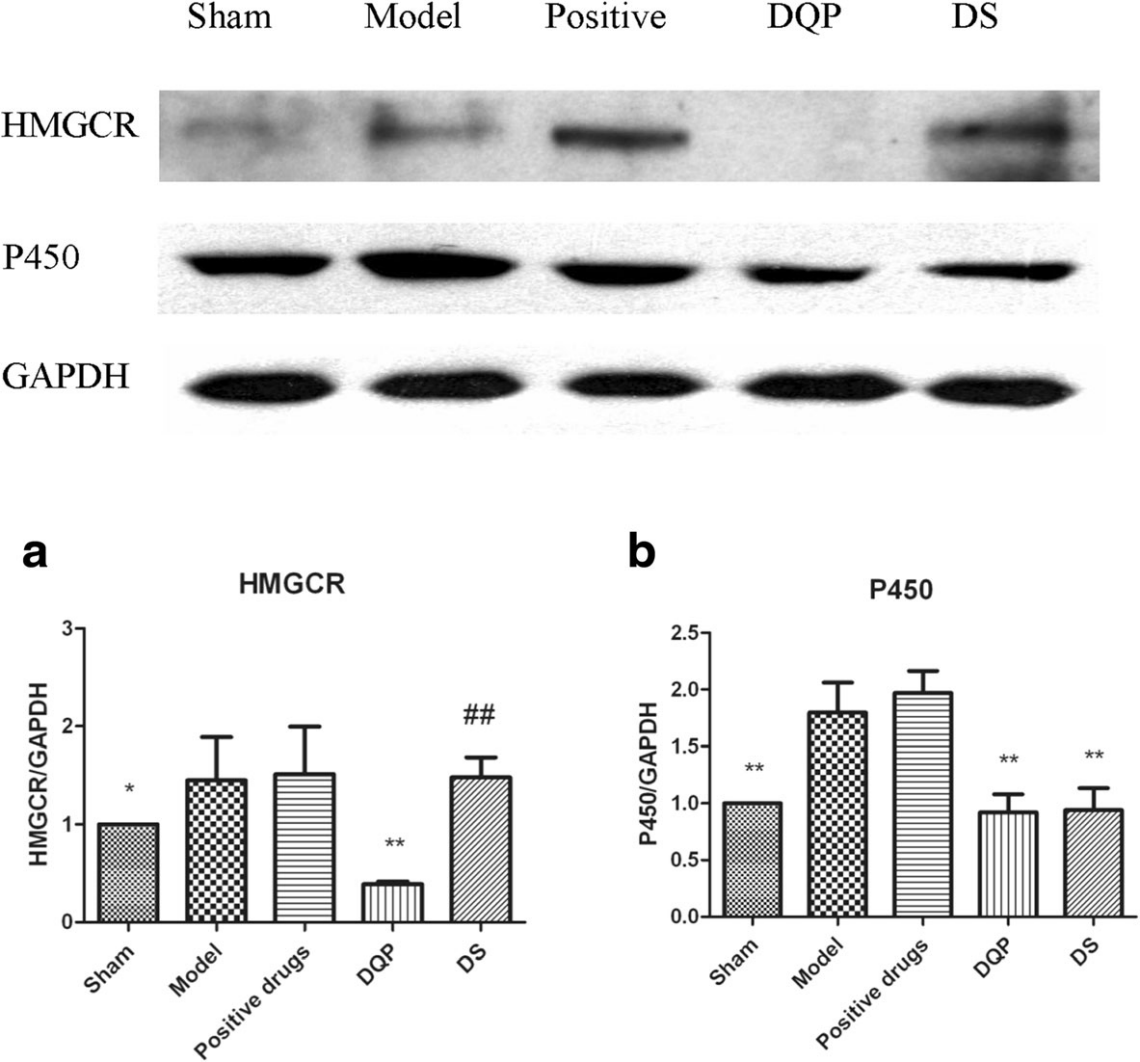
Expressions of HMGCR and P450 in different groups. **a** Semi-quantitative expressions of HMGCR in different groups. **b** Semi-quantitative expressions of P450 in different groups. *n* = 6,* v.s. model group, *P* < 0.05; ** v.s. model group, *P* < 0.01; ^##^ v.s. DQP group, *P* < 0.01

HMGCR is the rate-limiting enzyme in cholesterol synthesis. Inhibiting HMGCR is an effective way in reducing plasma level of cholesterol and HMGCR is the target of statins [19]. In model group, HMGCR was up-regulated compared with sham group (*P* < 0.05). After treatment with DQP, HMGCR was significantly reduced (*P* < 0.001). However, DS had no effect on expression of HGMCR (*P* > 0.05) (Fig. 2).

Cytochrome P450 is involved in lipid modification and cholesterol metabolism [20]. P450 level was increased in model group compared with that in the sham group (*P* < 0.01). After treatment with DQP and DS, P450 level was reduced significantly compared with that in the model group (*P* < 0.01), indicating that DQP and DS could affect lipid metabolism by regulating levels of P450 enzymes (Fig. 2). The inhibition effects of DQP and DS on P450 were similar.

### Effects of DQP and DS on PPARs, RXRA and PGC-1α

**Fig. 3:**
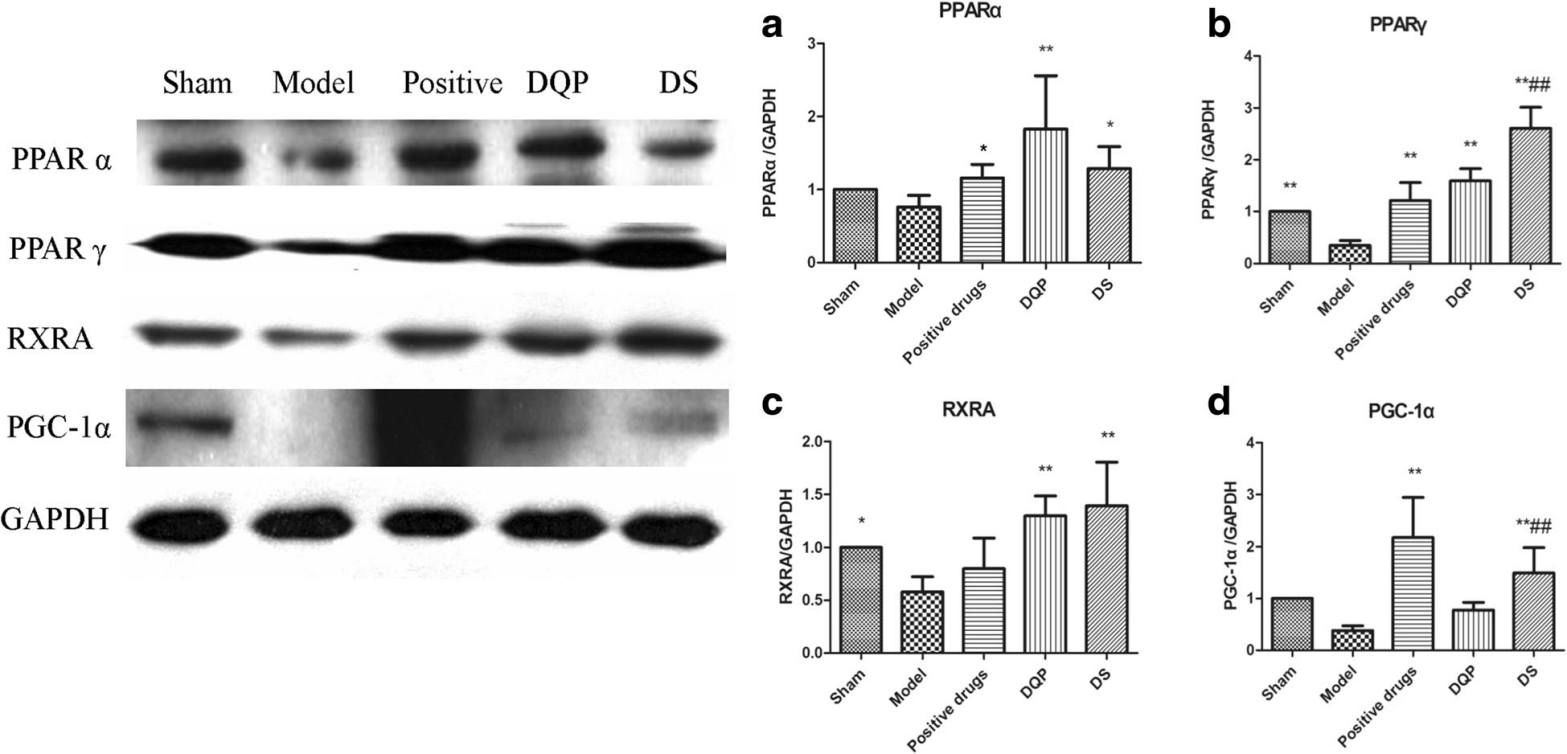
Expressions transcription regulators in five groups of rats. **a** Semi-quantitative expressions of PPAR-α in different groups. **b** Semi-quantitative expressions of PPAR-γ in different groups. **c** Semi-quantitative expressions of RXRA in different groups. **d** Semi-quantitative expressions of PGC-1α in different groups. *n* = 6, * v.s. model group, *P* < 0.05; ** v.s. model group, *P* < 0.01, ## v.s. DQP group, *P* < 0.01

PPARs are the master transcription factors in energy metabolism [21, 22]. PPARα can bind with retinoic acid receptor alfa (RXRA) to form heterodimer, promoting transcription of fatty acid oxidation genes [23]. PGC-1α is a transcriptional coactivator that regulates the genes involved in energy metabolism [24, 25]. Expression changes of these factors in different groups of rats were detected. Western blot showed that PPARα, PPARγ, RXRA and PGC-1α were down-regulated by 24.25% (*P* > 0.05), 64.8% (*P* < 0.01), 42.09% (*P* < 0.05) and 61.97% (*P* < 0.05) in model group compared with those in sham-operated group, respectively. After treatment with DQP, expressions of PPARα, PPARγ, RXRA and PGC-1α were up-regulated by 141.31% (*P* < 0.001), 352.54% (*P* < 0.001), 123.71% (*P* < 0.001) and 103.41% (*P* < 0.05) in DQP group compared with those in model group, respectively. DS could also up-regulate expressions of these factors. PPARα, PPARγ, RXRA and PGC-1α were increased by 69.66% (*P* < 0.05), 640.89% (*P* < 0.001), 140.08% (*P* < 0.001) and 289.80% (*P* < 0.001) in DS group compared with those in model group, respectively (Fig. 3). The effects of DS on PPARγ, RXRA and PGC-1α were more remarkable compared with those of DQP, whereas DQP showed better effect in increasing expression of PPARα than DS.

### Effects of DQP and DS on PI3K/AKT and ERK1/2

**Fig. 4:**
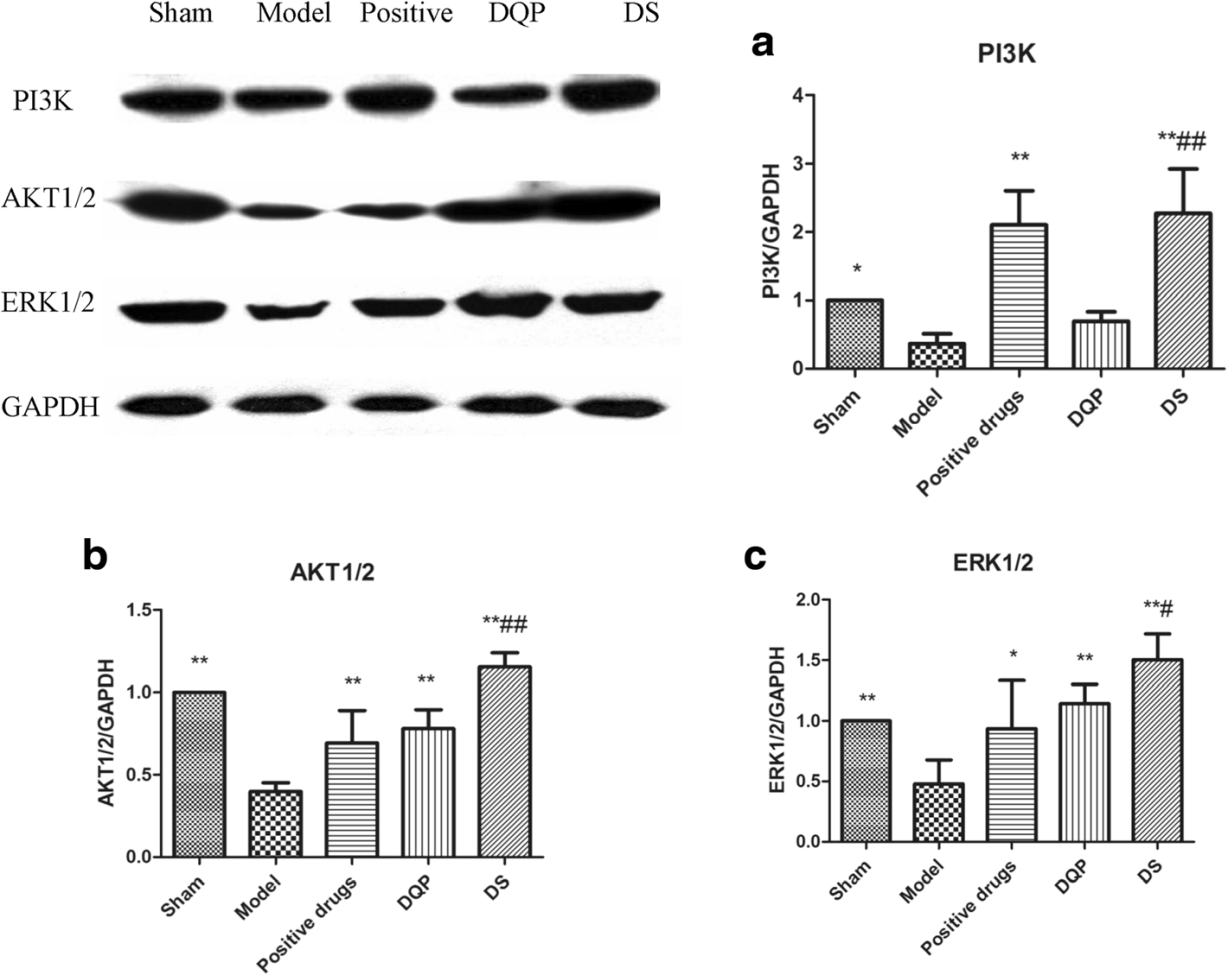
Expressions of PI3K, AKT1/2 and ERK1/2 in different groups. **a** Semi-quantitative expressions of PI3K in different groups. **b** Semi-quantitative expressions of AKT1/2 in different groups. **c** Semi-quantitative expressions of ERK1/2 in different groups. * v.s. model group, *P* < 0.05; ** v.s. model group, *P* < 0.01; ## v.s. DQP group, *P* < 0.05; ## vs DQP group, *P* < 0.01

PI3K/AKT and mitogen-activated protein kinase (MAPK)/ERK1/2 are important cardio-protective survival pathways [26]. Expressions of PI3K and AKT1/2 were down-regulated by 63.41% (*P* < 0.05) and 60.23% (*P* < 0.001) in model rats compared with those in sham-operated rats, indicating that PI3K/ATK pathway was inhibited in ischemic heart tissue. Expression of ERK1/2 was also suppressed in model group compared to sham-operated group (Fig. 4). Treatments with DQP or DS could increase expressions of PI3K/AKT and ERK1/2, re-activating the signaling pathways. Compared with model group, expressions of PI3K, AKT1/2 and ERK1/2 in DQP group were up-regulated by 89.62% (*P* > 0.05), 96.39% (*P* < 0.001) and 138.67% (*P* < 0.001), respectively. In DS group, expressions of PI3K, AKT1/2 and ERK1/2 were up-regulated by 521.05% (*P* < 0.001), 189.82% (*P* < 0.001) and 213.96% (*P* < 0.001), respectively. The effects of DS on PI3K/Akt and ERK1/2 were more remarkable than DQP, suggesting that the cardio-protective effects of DS might be mediated by activating PI3K/Akt and ERK pathways (Fig. 4).

## Discussion

In this study, we successfully induced rat model of ischemic coronary heart disease. Based on this model, we investigated the pharmacological mechanisms of DQP and its major components DS in the treatment of CHD. In the drug dosages we applied, cardiac functions were significantly improved as evidenced by reduced LVED and increased FS, suggesting that the current dosages were effective. Our major findings are as follows: 1) Both DQP and DS could exert cardioprotective effects under ischemic conditions. Heart functions were reserved in DQP and DS groups. 2) DQP and DS improved fatty acid oxidation in ischemic heart by up-regulating molecules involved in fatty acids transportation and uptake. 3) Both DQP and DS could up-regulate expressions of PPARα, PPARγ and RXRA. DQP was a better agonist of PPARα than DS, whereas the effects of DS on PPARγ, RXRA and PGC-1α were greater than those of DQP. 4) Expressions of PI3K/Akt and ERK1/2 were activated by DQP and DS. These results suggest that DQP and DS improve cardiac energy metabolism by up-regulating transcription factors such as PPARα, PPARγ and PGC-1α, thus providing cardioprotective effects under ischemic conditions. The effects of DQP and DS are comparable, indicating that DS are the major effective components in regulating energy metabolism.

Our previous studies illustrated that DanQi pill could regulate fatty acids metabolism in ischemic heart tissue. Not only can dyslipidemia cause coronary heart disease but coronary artery ligation can also induce dyslipidemia [9]. In this study, we re-confirmed that DQP could regulate components of apolipoprotein and key molecules involved in lipid transportation and uptake in ischemic heart model. Epidemiological studies showed that levels of HDL are inversely correlated with risk of cardiovascular events, whereas LDL levels are directly related to rate of these events [27, 28]. HDL protects against cardiovascular disease by regulating cholesterol efflux from tissues and modulating inflammation [29]. ApoA-1, the major protein component of HDL, is largely responsible for reverse cholesterol transport through the macrophage ATP-binding cassette transporter ABCA1 [30, 31]. Our study showed that expressions of ApoA-1 and ABCA1 were down-regulated in model group and treatments with DQP and DS up-regulated their expressions.

Several key molecules are involved in fatty acids transport and uptake by myocardiocyte. Fatty acid translocase/cluster of differentiation protein (CD36), a class of membrane protein, facilitates transport of long chain fatty acid into myocardiocyte [31, 32]. Plasma fatty acids are translocated into cardiac myocytes by FABP4 on the membrane and further transported into mitochondria by CPT-I. CPT-1A is one of the most abundant subtypes of CPT-I, which is the key enzyme in fatty acid oxidation process [33]. Fatty acid transport protein 4 (FATP4) is also involved in long- and very long-chain fatty acid uptake and facilitate acyl-CoA synthesis [34, 35]. These key molecules were down-regulated in model rats and treatments with DQP and DS significantly improved their expressions, suggesting that the lipid transport and uptake pathways could be regulated by DQP and DS. Furthermore, DQP could inhibit synthesis of cholesterol by reducing expression of HMGCR. DS didn’t seem to have effect on HMGCR, indicating that the inhibitory effect on cholesterol synthesis may be mediated by components other than Salvianolic acids or Panax notoginseng saponins.

Fatty acids oxidation provides the majority of energies needed for cardiac contraction and this process is tightly regulated by transcription factors. We further investigated the effect of DQP and DS on transcriptional regulation of energy metabolism. The PPAR family is a class of transcription factors that regulate cardiac fuel metabolism at the gene expression level. The three PPAR family members (α, β/δ and γ) are uniquely suited to serve as transducers of developmental, physiological, and dietary cues that influence cardiac fatty acid and glucose metabolism [22]. The principal transcriptional regulator of fatty acid oxidation (FAO) enzyme genes is PPARα [20]. PPARγ is a primary target for thiazolidinedione-structured insulin sensitizers like pioglitazone and rosiglitazone employed for the treatment of type 2 diabetes mellitus [36]. PGC-1α is transcriptional coactivator of the PPARs and is a key player in the control of myocardial metabolism. In cardiac myocytes, activation of PGC-1α drives a strong induction of PPARα target genes encoding FAO enzymes [14]. PGC-1α also coactivates other transcription factors, including estrogen-related receptors and the nuclear respiratory factor 1, to stimulate mitochondrial biogenesis and enhance expression of components of the electron transport chain [37]. In ischemic heart disease, the expression and/or DNA binding activity of the PPARα-RXR complex is markedly diminished by hypoxia, which results in decreased expression of genes encoding enzymes involved in mitochondrial FAO and oxidative phosphorylation pathways [38, 39]. The role of PPARs in ischemia is controversial. Some studies found that chronic activation of PPARα is detrimental to cardiac recovery after ischemia and PPARγ activator was associated with increased mortality post-myocardial infarction in rats [40–42]. In our study, we found that expressions of PPARα, PPARγ, RXRA and PGC-1α were all down-regulated in ischemic model group. After treatments with DQP or DS for 28 days, the expressions of these transcriptional factors were up-regulated compared with those in the model group. DQP showed better effect on PPARα, whereas DS seemed to be more effective in up-regulating PPARγ and PGC-1α. The different effects exerted by DQP and DS may be due to complex interactions of different components in DQP compound [43]. These results demonstrated that up-regulating transcription factors targeting energy metabolism are cardio-protective under ischemic conditions. Components of DS may serve as ligand activators of PPARs. Besides DS, other compounds in DQP may also activate expressions of PPARα, as DQP improved PPARα expression in a more remarkable way. The exact mechanism by which DQP up-regulates PPARs warrants further studies. The active compounds in DQP, other than DS, also need further investigation.

The potential effects of DQP and DS on signaling pathway were also studied. It is widely accepted that phosphatidylinositol 3-kinase (PI3K) signaling to Akt is an important regulator of many cellular processes including proliferation, apoptosis and nitric oxide synthesis [44]. PI3K also increases cardiac fatty acid oxidative capacity [45]. Activation of PI3K/Akt pathway has been shown to play a key role in myocardial preconditioning [46]. PI3K signaling cascade reduces myocardial damage after ischemia by recruiting multiple endogenous cardioprotective pathways. ERK1/2 belongs to the MAPKs family and activation of ERK1/2 in cardiac myocytes serves as a defense mechanism against ischemic stress stimuli [47]. In this study, we found that expressions of PI3K, Akt1/2 and ERK1/2 were all reduced in model rats, and administrations of DQP and DS increased the expressions of these molecules, indicating that activation of PI3K/Akt and ERK1/2 is protective under ischemic conditions and DQP could activate the signaling pathway.

## Conclusions

DS are the major effective components of DQP in improving energy metabolism in ischemic heart disease model. The effects may be mediated by regulating transcriptional factors such as PPARs, RXRA and PGC-1α. Further study into the pharmacological mechanisms of Salvianolic acids and Panax notoginseng will provide insight into the discovery of new drugs targeting PPARs and PGC-1α and new strategies in the management of cardiovascular diseases.

## Abbreviations

ABCA1: ATP-binding cassette transporter
ApoA-I: Apolipoprotein A-I
CD36: Cluster of differentiation 36
CHD: Coronary heart disease
CPT-1A: Carnitine palmitoyltransferase 1A
DQP: DanQi pill
DS: Panax notoginseng saponins
EF: Ejection fraction
ERK1/2: Extracellular signal-regulated kinase
FABP: Fatty acid binding protein
FS: Fractional shortening
GAPDH: Glyceraldehyde-3-phosphate dehydrogenase
HDL: High density lipoprotein
HMGCR: 3-hydroxy-3methyl-glutaryl-CoA reductase
LAD: Left anterior descending
LCAT: Lecithin cholesterolacyltransferase
LDL: Low density lipoprotein
LEVSv: Left ventricular end-systolic volume
LVEDd: Left ventricular end-diastolic diameter
LVEDv: Left ventricular end-diastolic volume
LVESd: Left ventricular end-systolic diameter
MAPK: Mitogen-activated protein kinase
PGC-1α: Peroxisome proliferator-activated receptor gamma coactivator-1α
PI3K: Phosphatidylinositol 3-kinase
PNS: Panax Notoginseng Saponins
PPAR: Peroxisome proliferator-activated receptor family
RT-PCR: Reverse transcriptase polymerase chain reaction
RXRA: Retinoic acid receptor alfa
SA: Salvianolic acid
SDS: Sodium dodecyl sulphate
TCM: Traditional Chinese medicine
